# 4360 Black Bone MRI from the Lab to Clinical Practice: Eliminating Radiation Exposure in Reconstructive Surgery Patients

**DOI:** 10.1017/cts.2020.325

**Published:** 2020-07-29

**Authors:** Marissa Suchyta, Christopher Hunt, Waleed Gibreel, Diya Sabbagh, Kryzysztof Gorny, Samir Mardini

**Affiliations:** 1 Mayo Clinic

## Abstract

OBJECTIVES/GOALS: Virtual surgical planning and 3D printing enable streamlined surgeries and increased complexity. These technologies, however, require CT scans and radiation exposure. This project’s goal is to optimize and demonstrate the accuracy of Black Bone MRI for surgical planning in reconstructive surgery. METHODS/STUDY POPULATION: Four common craniofacial surgeries were planned and performed on cadaver specimens (maxillary advancement, orbital floor reconstruction with patient-specific implants, cranial vault reconstruction, and fibular free flap reconstruction of the mandible). For each surgical procedure, ten cadaver heads were used. Five of each surgery were planned and 3D printed guides were created utilizing Black Bone MRI versus five with CT scans. Following mock surgeries, all specimens underwent a post-operative CT scan. 3d reconstruction was performed and surgical accuracy compared to the plan was assessed using GeoMagic Wrap, assessing average post-operative deviation from plan. RESULTS/ANTICIPATED RESULTS: In all surgeries, guides created from Black Bone MRI demonstrated high accuracy to surgical plan. Average osteotomy (cut) deviation from plan was not statistically significantly different when Black Bone MRI was used compared to CT scans for planning and guide creation in the wide variety of craniofacial surgeries performed. The average deviation of post-operative anatomy from pre-operative plan was also not statistically significant when Black Bone MRI versus CT scans were utilized in the surgeries. These results then enabled the translational application of this technology clinically, and we demonstrate a clinical reconstructive craniofacial case planned utilizing Black Bone MRI. DISCUSSION/SIGNIFICANCE OF IMPACT: This study demonstrates that virtual surgical planning and 3d surgical guide creation can be performed using Black Bone MRI with comparable accuracy to CT scans in a wide variety of craniofacial procedures. This could dramatically reduce radiation exposure for patients. The successful segmentation, virtual planning, and 3d printing of accurate guides from Black Bone MRI demonstrate potential to change the pre-operative planning standard of care. This project, overall, also demonstrates the development of new solutions to advance clinical care, thus serving as an example of moving translational science from a concept to the operating room.

